# Exergaming With Beat Saber: An Investigation of Virtual Reality Aftereffects

**DOI:** 10.2196/19840

**Published:** 2020-10-23

**Authors:** Ancret Szpak, Stefan Carlo Michalski, Tobias Loetscher

**Affiliations:** 1 University of South Australia Adelaide Australia

**Keywords:** virtual reality, motion sickness, exercise, sedentary behavior, depth perception

## Abstract

**Background:**

Virtual reality (VR) exergaming has the potential to target sedentary behavior. Immersive environments can distract users from the physical exertion of exercise and can motivate them to continue exergaming. Despite the recent surge in VR popularity, numerous users still experience VR sickness from using head-mounted displays (HMDs). Apart from the commonly assessed self-reported symptoms, depth perception and cognition may also be affected. Considering the potential benefits of VR exergaming, it is crucial to identify the adverse effects limiting its potential and continued uptake.

**Objective:**

This study aims to investigate the consequences of playing one of the most popular VR exergames for 10 and 50 min on aspects of vision, cognition, and self-reported VR sickness.

**Methods:**

A total of 36 participants played an exergame, called Beat Saber, using an HMD. A repeated measures within-subject design was conducted to assess changes in vision, cognition, and well-being after short (10 min) and long (50 min) durations of VR exposure. We measured accommodation, convergence, decision speed, movement speed, and self-reported sickness at 3 test periods—before VR, immediately after VR, and 40 min after VR (late).

**Results:**

Beat Saber was well tolerated, as there were no dropouts due to sickness. For most participants, any immediate aftereffects were short-lived and returned to baseline levels after 40 min of exiting VR. For both short and long exposures, there were changes in accommodation (*F*_1,35_=8.424; *P*=.006) and convergence (*F*_1,35_=7.826; *P*=.008); however, in the late test period, participants returned to baseline levels. Measures on cognition revealed no concern. The total simulator sickness questionnaire (SSQ) scores increased immediately after VR (*F*_1,35_=26.515; *P*<.001) and were significantly higher for long compared with short exposures (*t*_35_=2.807; *P*=.03), but there were no differences in exposure duration in the late test period, with scores returning to baseline levels. Although at a group level, participants’ sickness levels returned to baseline 40 min after VR exposure, approximately 14% of the participants still reported high levels of sickness in the late test period after playing 50 min of Beat Saber. We also showed that the participants who experienced a high level of sickness after a short exposure were almost certain to experience a high level of symptoms after a longer exposure.

**Conclusions:**

Irrespective of the duration of exposure, this study found no strong evidence for adverse symptoms 40 min after exiting VR; however, some individuals still reported high levels of VR sickness at this stage. We recommend that users commit to a waiting period after exiting VR to ensure that any aftereffects have deteriorated. Exergames in HMDs have the potential to encourage people to exercise but are understudied, and the aftereffects of exergaming need to be closely monitored to ensure that VR exergames can reach their full potential.

## Introduction

### Background

Exergaming combines exercise and gameplay into a virtual environment with the intention of promoting greater physical activity among users. Exergaming is a valid method for targeting sedentary behaviors in children, adolescents [1], and adults [2,3]. The physiological health benefits of exergames are comparable with exercise such as running and aerobic dancing [1]. Several studies show that some people get greater enjoyment and feel more positive toward exergaming compared with other forms of physical exercise [1,4,5]. Greater enjoyment of exercise and adherence are linked to the psychological experience of flow during physical activities [6]. Exergaming facilitates the flow experience by providing an opportunity to become easily absorbed in the goals and challenges in the game [7,8]. A combination of physiological and psychological benefits of physical activities makes exergaming an appealing strategy to encourage sedentary people to exercise.

A potentially promising avenue to boost the effectiveness of exergaming is virtual reality (VR). One main reason why exergaming is so successful is that it can distract the user from the physical exertion of exercise [4,9]. Users become deeply involved in the game (flow state) and are more motivated to continue engaging in the tasks and narrative of the game even if it requires physical and mental effort. When it comes to involvement and absorption, VR excels in this domain. By offering a combination of realistic 3D environments, immersive 360˚ spaces, and body tracking, these features allow users to feel like they exist in the virtual world and are ready to face all the challenges and experiences it affords. Typically, presence is not a prerequisite for exergames but can provide added benefits [9]. However, in VR, presence is the cornerstone of a successful virtual experience, with greater presence leading to better task performance and stronger physiological responses compared with other screen-based activities [10-12]. Similarly, immersion and presence in VR exergames play a major role in the motivation of users and their continued engagement [9].

Reviews of VR exergames are an important indicator of user attitudes and receptivity of using VR experiences as a motivator to exercise. A recent study [9] examined reviews of popular VR exergames to identify the positive and negative factors contributing to their continued engagement. Generally, users said that they enjoyed immersive exergames that distracted them from the intensity of the exercise and felt that VR games, such as Beat Saber, provided a similar level of physical activity they would typically get from exercising in the real world [9]. Negative reviews identified *motion sickness* as a hindrance to their in-game performance and perceived enjoyment [9].

*Motion sickness*, or VR sickness, has plagued VR since its early days. While the VR industry has seen a surge in accessibility, greater reliability, and lower prices even for casual consumers, many users are still affected by symptoms of VR sickness [13-15]. High levels of VR sickness symptoms such as nausea, disorientation, and visual disturbances have been estimated to result in an average dropout rate of 15.6% [14]. VR sickness has been shown to decrease or break presence [10,11], impact motivation and enjoyment [9,16], and influence task performance [17-19]. Apart from the commonly assessed self-reported symptoms, depth perception and cognition may also be affected when users experience VR sickness [15,20,21]; however, little is known about these aftereffects. Considering the potential benefits of serious VR gaming, it is crucial to better understand the adverse effects that limit its potential and continued uptake.

There is no consensus on the etiology of VR sickness [22-24]. One prominent theory is the mismatch between virtual and real worlds. A visual-vestibular conflict can be introduced in VR when the visual experience does not match the physical or bodily experience [13,25]. The integration of visual and vestibular (ie, bodily) sensations plays a fundamental role in an individual's ability to move about and interact with their environment [26,27]. If there is a conflict between visual and vestibular sensations relayed back to the brain, an individual may experience a disturbance in sensory integration leading to the occurrence of motion sickness symptoms such as nausea or disorientation. An individual may also experience an increase in oculomotor symptoms resulting from vergence-accommodation conflicts from HMDs [15,20,28]. Vergence and accommodation are essential oculomotor functions that facilitate the accurate use of depth cues [15]. It is unclear whether vergence-accommodation conflicts are responsible for VR sickness in certain individuals or whether it compounds the severity of sickness symptoms.

Considering the factors that induce VR aftereffects, it is clear that both content and device characteristics play a key role in the onset and progression of VR sickness. To address the role of content, the Oculus store (Facebook Technologies LLC, 2012) includes comfort ratings for most VR games, which are either rated *comfortable*, *moderate*, or *intense* to warn users of potential adverse side effects [29]. These ratings are based on the amount of camera movement, user motion, and occasional disorienting content that can be perceived under a typical experience. However, these ratings do not factor in the length of the experience and how longer exposures may contribute to VR aftereffects [14,30]. Similarly, device manufacturers of HMDs address VR sickness or side effects in their instruction manuals and make safety recommendations for VR usage [31-33]. These safety guidelines are reliant on the self-assessment of their users to gauge the symptoms of VR sickness. Recommendations for VR usage durations range from 30 min to hour-long exposures, and some devices do not specify but leave it up to the user to decide [31-33]. As VR is highly immersive, users can easily lose track of time, resulting in longer exposure times. Research shows that longer exposures lead to a higher prevalence of serious self-reported symptoms [14,30]. Publicly available research supporting manufacturer recommendations for VR exposure durations is limited.

Research investigating the impact of VR exposure duration primarily focuses on self-reported symptoms and shows that the length of time spent in VR is a critical factor in the development and severity of aftereffects [14,34,35]. However, a recent review suggests that the relationship between VR content and exposure duration may not be straightforward [14]. The authors found that lower self-reported symptoms were recorded for exposures less than 10 min compared with longer VR studies between 10 and 20 min. Intriguingly, studies that were longer than 20 min on average reported less severe symptoms than studies between 10 and 20 min. The authors suggested that the distribution of the types of VR content (ie, 360˚ videos, game, minimalist, scenic) in each of the time categories may have contributed to this nonlinear pattern of results [14]. There are few studies that directly compare aftereffects in the same content with shorter and longer exposure durations. Hence, it is difficult to answer the question of whether users who experience short exposures to a particular VR content will experience similar or worse symptoms for longer exposures with the same content.

Another important safety issue to consider is the duration of aftereffects after exiting VR and the impact the duration of aftereffects may have on recovery. There is a gradual degradation of VR sickness symptoms, and it is often recommended that users experiencing symptoms stop VR and wait until they have recovered [32,36]. The duration of these symptoms may vary depending on the time spent in VR and the initial severity of symptoms [34,37]. A recent review suggests that although there is an increase in symptoms with exposure durations, the persistence of aftereffects varies considerably from short periods of time (10 min) to longer periods (4 hours) [34]. Furthermore, recovery from aftereffects may take longer if a user experiences severe symptoms [34].

### Objectives

With the growth in VR exergaming and entertainment, an increasing number of people will likely experience symptoms from longer exposures. Using a popular VR exergame, this study addresses the influence of exposure durations on a user’s well-being, and on aspects of vision and cognition. One of the most successful commercial VR exergames is called Beat Saber, with over 2 million copies sold worldwide [38]. According to the Virtual Reality Institute of Health and Exercise [39], the time spent playing Beat Saber is comparable with the energy spent playing tennis in the real world. Therefore, Beat Saber provides a compelling test case for studying the aftereffects of VR exergaming. This study examined VR aftereffects from exergaming through long (50 min) and short (10 min) exposure durations. For both exposure durations, measures of near-point convergence and accommodation, reaction times, and self-reported symptoms were taken before, immediately after VR, and 40 min after VR (late measurements).

## Methods

### Participants

A power analysis [40] was performed using a medium effect size (0.54) from the average effect sizes for vision and cognitive tests in Szpak et al [11]. Accordingly, the power analysis for a one-sample *t* test (difference from constant) with α=.05 and 1–ß=.80 suggested that a minimum sample of 29 participants was required. We tested all participants who signed up when the study was advertised, which resulted in a total of 44 participants.

A total of 44 English-speaking participants were recruited and provided informed consent for participation. Participants were reimbursed AUD $20 (USD $14.6) per hour of participation. One participant withdrew (*male*) from the study, which was not due to VR sickness, and another 7 participants (*male*=5; *female*=2) were excluded because of stereoacuity of 100 arcseconds or worse. The remaining 36 participants (*male* 21; *female*=15) were included in the main analyses (mean age 20.55, SD 2.29 years). Of these 36 participants, 17 (47%) self-reported to play computer/console games on a weekly or daily basis, 50% played on a monthly basis or less, and 1 person did not specify. The average total Motion Sickness Susceptibility Questionnaire Short−form (MSSQ-short) score was relatively low at 8.97 (SD 6.14) compared with other studies and norm data [25,26]. The Human Research Ethics Committee at the University of South Australia granted ethics approval for this study.

### Materials and Apparatus

#### Virtual Reality Setup

A commercially available HTC Vive Pro HMD was used to administer a VR rhythm exergame—*Beat Saber* (developed by Beat Games). Beat Saber was selected because it is a best-selling exergame with a large user base and it offers a high-quality, responsive, and enjoyable game that participants could engage with for at least 60 min. A high-end laptop with an Intel Quad-Core i7−7820HK processor at 2.90 GHz, 16 GB RAM, and an Nvidia GeForce GTX 1080 8 GB graphics card, which ensures that participants experience the game at optimal performance. Using motion tracking, Beat Saber simulates handheld controllers as light sabers, whereby users slash targets and must actively avoid incoming obstacles to the rhythm of the beat. The game provided haptic, auditory, and performance feedback, thereby giving participants an immersive experience.

#### Visual Measures

Stereo vision, near vision, and distance vision were measured to screen participants’ visual and stereoacuity. The Snellen [41] and Fonda-Anderson [42] charts were used to assess distance vision and near vision, respectively. The Butterfly Stereo Acuity test (Vision Assessment Corporation, 2007) was utilized to ensure participants could see the virtual environment correctly. Furthermore, accommodation and vergence were measured to investigate changes in participants' depth perception and vision. The Royal Air Force (RAF) near-point rule [43] was used to assess the near point of convergence and the near point of accommodation before and after VR exposure. The RAF near-point rule is composed of a 500-mm ruler-like square tube with a slider attachment bracketing a 4-sided rotating cuboid. In this study, we used only 2 of the 4 sides: the Times Roman typeface to measure accommodation and a small black dot to measure convergence. At one end of the RAF rule, there is a plastic 60-mm V-shaped cheek rest to comfortably sit on a participant's cheek and fit around his or her nose [43]. Participants’ accommodation and vergence measurements were measured in millimeters.

#### Cognitive Measures

The CANTAB 5-choice reaction time task (RTI) was administered on an iPad 2 using the CANTAB app [44]. The CANTAB version of the 5-choice RTI focuses on measuring participants’ speeded responses so that movement and cognitive factors are dissociable. The 5-choice RTI requires a participant to monitor 5 locations. The RTI consists of a circle (button) on the lower half of the screen and 5 circles on the top of the screen. The participant must press the button located at the bottom of the screen and wait for a yellow dot to appear in any of the 5 circles on the top of the screen. When a yellow dot appears, the participant must release the button and touch the yellow dot (target stimulus) as quickly as possible. The reaction time for this task comprises 2 components: decision and movement speed. In this study, decision speed is the median duration from the time the target stimulus appeared to the moment the participant released the button. The movement speed is the median duration of the release of the button to the touch of the target stimulus. Only correct responses were used to calculate these components.

#### Self-Report Questionnaires

The MSSQ-short was employed to measure how susceptible participants are to motion sickness [45,46]. The MSSQ-short [46] has a high correlation with the long version (*r*=0.93) [45,46] and has an internal consistency of α=.91 [46].

The Simulator Sickness Questionnaire (SSQ) [47] is the most commonly used questionnaire in simulator and VR studies [14,25] and has a good internal consistency α*=.87* [48]. The SSQ was used in this study to measure self-reported symptoms of VR sickness [47,49]. The SSQ comprises a 16-symptom inventory with a 4-point rating scale from 0 (none) to 3 (severe). Each symptom cluster was divided into 3 categories: nausea, oculomotor, and disorientation. The nausea cluster comprises 7 symptoms associated with feelings of stomach sickness, such as increased salivation, burping, and stomach awareness. The oculomotor cluster consists of 7 symptoms related to eyestrain, fatigue, and focus. The disorientation cluster includes 7 symptoms related to dizziness and vertigo. The 3 subscales include overlapping symptoms from the other subscales. Raw scores are weighted differently for total scores and the subscales [12,38,39].

### Procedure

First, each participant was given verbal instructions and guided through the consent process. All participants were screened to ensure that they had normal vision and stereoacuity using the Butterfly Stereo Acuity test, the Snellen chart, and the Fonda-Anderson chart. Participants completed several questionnaires that included questions regarding demographics (eg, age, gender, handedness), gaming experience, vision history, and motion sickness susceptibility (MSSQ-short).

At baseline, participants’ accommodation and convergence were measured with the RAF rule, and they also completed the CANTAB RTI on an iPad and paper-based SSQ. Participants were then immersed in a VR exposure that required them to play Beat Saber using the HTC Vive Pro HMD. During this time, participants had either a 10-min or 50-min exposure in the VR. All participants completed both a 10-min and 50-min exposure on 2 separate days. The order of exposure duration was counterbalanced across participants.

Participants were instructed to play the in-game tutorial to understand how the game is played. The tutorial was completed only during the participant's first intervention period. Once the tutorial was completed, the researcher explained that participants had an opportunity for *free play*, that is, they could play the game at their leisure and choose any song and difficulty of their choice.

**Figure 1:**
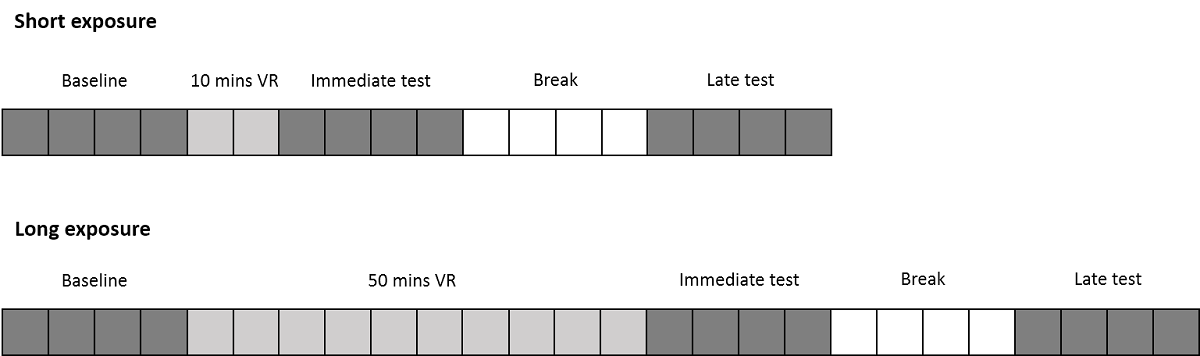
The study design of both days of participation. One square represents 5 min. Dark gray squares represent assessment periods, light gray squares represent virtual reality (VR) exposure, and white squares show when participants took a 20-min break. The order of short and long VR exposures on days 1 and 2 was counterbalanced between participants.

Immediately after each VR exposure, participants completed the same measures they completed before VR in the following order: accommodation, vergence, RTI, and SSQ. Participants then had a 20-min break and, finally, completed these same measures in a late test period 40 min after VR exposure (Figure 1).

### Analyses

The following analyses used difference scores that were calculated for all measures to demonstrate the change from baseline to immediate or late measurements. All difference scores were analyzed with the repeated measures analysis of variance with test periods (immediate and late) and exposure durations (10 min and 50 min) as within-subject factors. Bonferroni corrections were employed for all planned pairwise post hoc comparisons. Furthermore, one-sample *t* tests were performed to determine whether the difference scores significantly changed from zero, that is, represent a significant difference from baseline (Table 1). Alluvial plots were generated from weighted SSQ scores and categorized from a modified version of Kennedy et al [49], resulting in the following 3 sickness levels: low (0-10), mid (>10 to 20), and high (>20).

## Results

Participants’ data are available on the Open Science Framework [50].

### Visual Measurements

**Figure 2:**
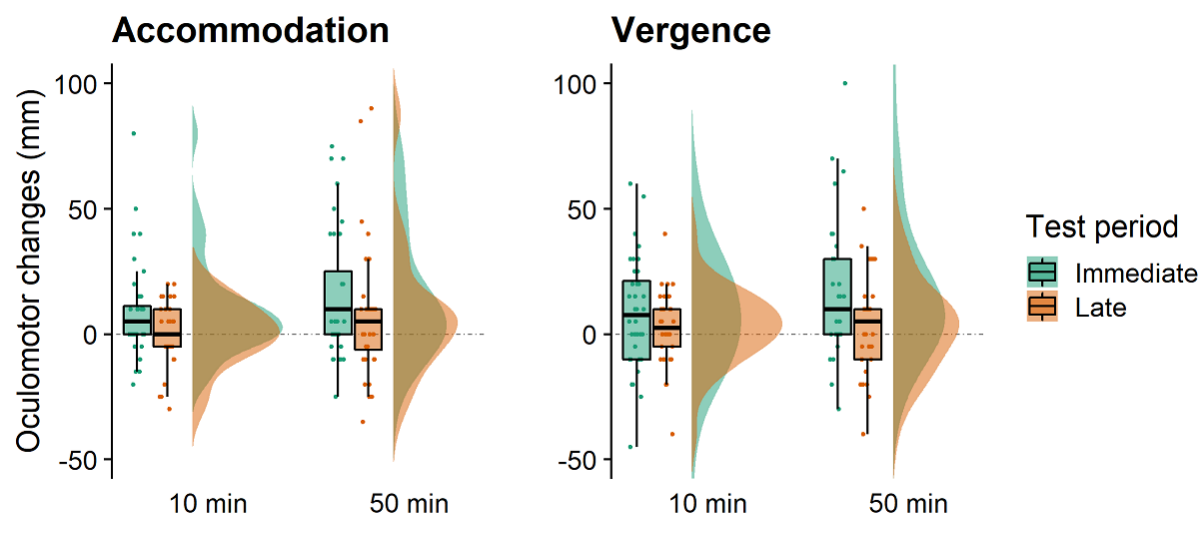
Raincloud plots for accommodation (left) and vergence (right) measures showing the different test periods and virtual reality exposure times. Positive and negative scores, respectively, indicate an increase (further) or decrease (nearer) change in accommodation or vergence from baseline measurements.

For accommodation measurements, there was a significant main effect of the test period (*F*_1,35_=8.424; *P*=.006; partial *η*^2^=0.194), with larger accommodation changes immediately after VR (mean 12.43, SE 3.06) compared with the late (mean 4.10, SE 1.92) measurement test period (Figure 2). There was no effect of the exposure duration (*F*_1,35_=2.974; *P*=.09; partial *η*^2^=.078), and the interaction was not significant (*F*_1,35_=0.035; *P*=.85; partial *η*^2^=0.001).

For convergence measurements, there was a significant main effect of test period (*F*_1,35_=7.826; *P*=.008; partial *η*^2^=0.183) with larger convergence changes immediately after VR (mean 13.68, SE 3.65) compared with the late (mean 5.00, SE 2.93) measurement (Figure 2). There was no effect of the exposure duration (*F*_1,35_=2.159; *P*=.15; partial *η*^2^=0.058), and the interaction was not significant (*F*_1,35_=1.334; *P*=.26; partial *η*^2^=0.037).

### Cognitive Measurements

The CANTAB 5-choice RTI captured both decision times and movement speeds. Accordingly, these components were analyzed separately to determine which aspect of the reaction speeds may be affected by VR.

**Figure 3:**
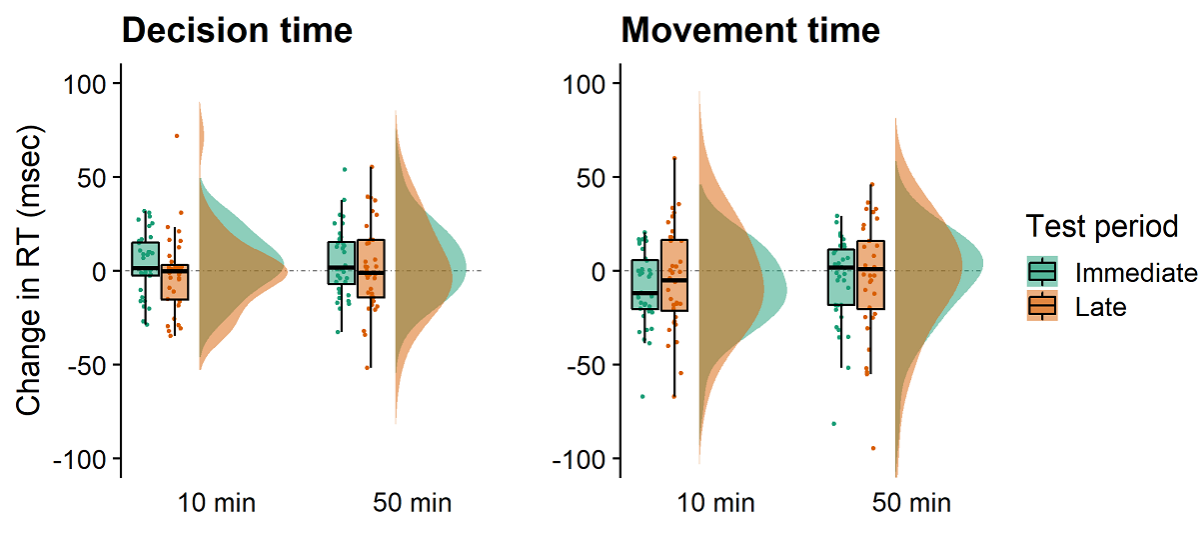
Raincloud plots for the cognitive 5-choice reaction time task showing reaction time (RT) difference scores for the different test periods and virtual reality exposure times. This task captures both decision speeds (left) and movement speeds (right). Positive and negative scores indicate the participant's RTs becoming slower or faster from baseline measurements. RT: reaction time.

Regarding decision times, there was a significant main effect of the test period (*F*_1,35_=4.671; *P*=.04; partial *η*^2^=.118) with larger changes (slower reaction times [RTs]) in the decision speed immediately after VR (mean 4.472, SE 2.229) compared with the late (mean .257, SE 2.638) measurements (Figure 3). There was no effect of the exposure duration (*F*_1,35_=0.440; *P*=.51; partial *η*^2^=0.012)*,* and the interaction was also not significant (*F*_1,35_=0.668; *P*=.42; partial *η*^2^=0.019).

Regarding movement times, there was no main effects of the test period (*F*_1,35_=1.506; *P*=.23; partial *η*^2^=0.041) or the exposure duration (*F*_1,35_=0.206; *P*=.65; partial *η*^2^=0.006)*,* and the interaction was not significant either (*F*_1,35_=0.784; *P*=.38; partial *η*^2^=0.022; Figure 3).

### SSQ

**Figure 4:**
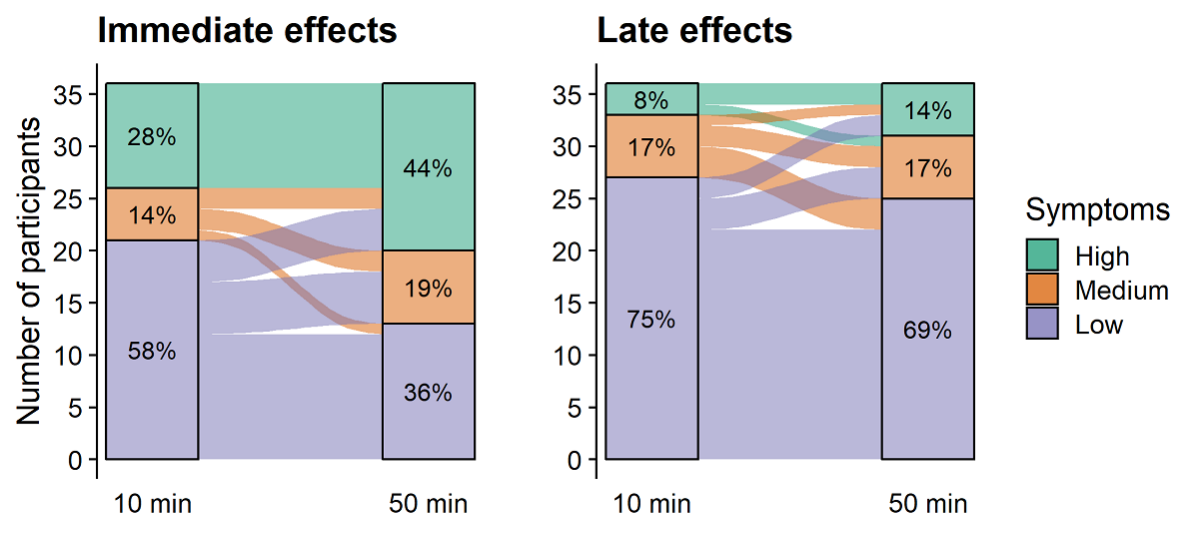
Alluvial plots showing how participants' flow from one virtual reality sickness category to another on the basis of exposure duration and test period. Bars indicate the percentage of participants who are in each of the high, mid, and low virtual reality sickness symptom categories for 10-min and 50-min exposures. Left and right panels show the flow of the categories for the immediate and late test period, respectively.

Total SSQ scores and subscales (nausea, oculomotor, and disorientation) were weighted according to Kennedy et al [47]. Difference scores were calculated from these weightings. Alluvial plots were created to visualize changes in SSQ symptom levels in participants across the different exposure times and test periods (Figure 4).

**Figure 5:**
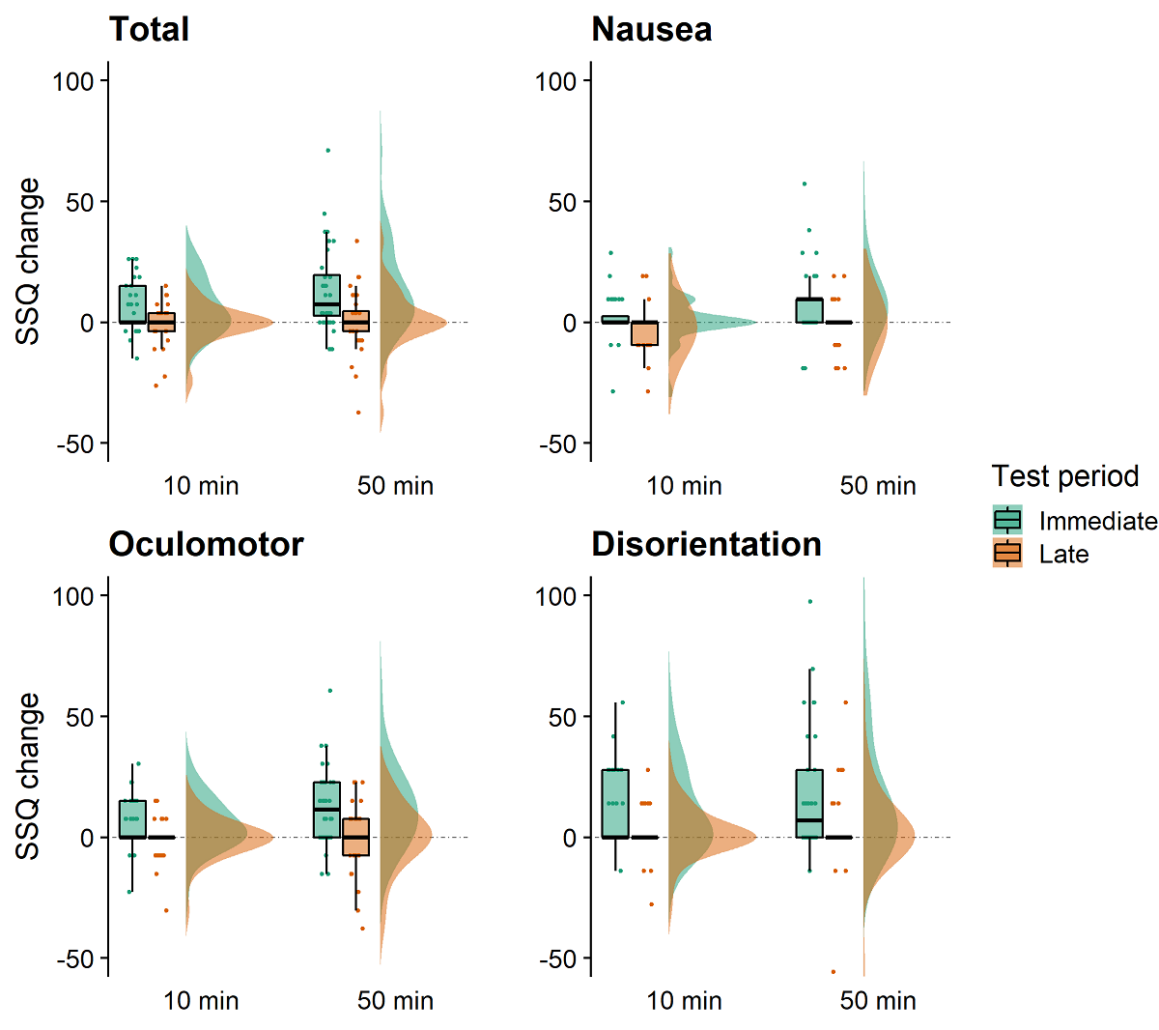
Raincloud plots for the total Simulator Sickness Questionnaire and subscale difference scores showing test periods and exposure duration. Positive and negative scores, respectively, indicate an increase and decrease in sickness symptoms compared with baseline. SSQ: Simulator Sickness Questionnaire.

For total SSQ difference scores, there was a significant main effect of the test period (*F*_1,35_=26.515; *P*<.001; partial *η*^2^=0.431), with larger changes immediately after VR (mean 9.869, SE 1.952) compared with the late measurement (mean –0.052, SE 1.272; Figure 5). There was also an effect of exposure (*F*_1,35_=4.816; *P*=.04; partial *η*^2^=0.121), with a 50-min VR exposure (mean 7.220, SE 2.038)*,* leading to larger changes than a 10-min exposure *(*mean 2.597, SE 1.280). The significant interaction (*F*_1,35_=4.738; *P*=.04; partial *η*^2^=0.119) was followed up with a number of post hoc paired *t* test comparisons. They showed that for 10 min of VR, immediate measurements (mean 6.026, SE 1.325) were significantly different from late measurements (mean −0.831, SE 1.325; t_35_=3.868; *P*=.002). Comparisons for 50 min of VR showed that immediate measurements (mean 13.713, SE 2.878) and late measurements (mean 0.727, SE 2.037) were also statistically different (t_35_=4.522; *P*<.001). A comparison of the immediate measurements between the 10 min and 50 min sessions of VR were also significantly different (t_35_=2.807; *P*=.03) from each other. However, the late measurements taken after 10 min and 50 min of VR were not significantly different (t_35_=0.675; *P*=.99).

For nausea difference scores, there was a significant main effect of the test period (*F*_1,35_=22.379; *P*<.001; *partial η*^2^=0.390), and the effect of the exposure duration (*F*_1,35_=4.549; *P*=.04; *partial η*^2^=0.115) and the interaction was also significant (*F*_1,35_=4.334; *P*=.045; *partial η*^2^=0.110; Figure 5). Paired *t* test comparisons showed that for 10 min of VR, immediate measurements (mean 1.590, SE 1.496) were significantly different from late measurements (mean −2.385, SE 1.441; t_35_=2.667; *P*=.048). Comparisons for 50 min of VR showed that immediate (mean 8.480, SE 2.336) and late measurements (mean −0.530, SE 1.419) were also statistically different (t_35_=4.261; *P*<.001). A comparison of immediate measurements between the 10 min and 50 min sessions of VR were also significantly different (t_35_=2.745; *P*=.04) from each other. However, the late measurements taken after 10 min and 50 min of VR were not significantly different (t_35_=0.827; *P*=.99).

For oculomotor difference scores, there was a significant main effect of the test period (*F*_1,35_=21.173; *P*<.001; partial *η*^2^=0.377) and the effect of the exposure duration failed to reach significance (*F*_1,35_=3.795; *P*=.06; partial *η*^2^=0.098); however, the interaction was significant (*F*_1,35_=4.817; *P*=.04; partial *η*^2^=0.121; Figure 5). Paired *t* test comparisons showed that after 10 min of VR, immediate measurements (mean 5.263; SE 1.677) were significantly different from late measurements (mean −0.632, SE 1.329; t_35_=3.500; *P*=.004). Comparisons for 50 min of VR showed that immediate (mean 12.002, SE 2.587) and late measurements (mean 0.211, SE 2.229) were also statistically different (t_35_=4.128; *P*<.001). A comparison of immediate measurements between the 10 min and 50 min sessions of VR were also significantly different (t_35_=2.652; *P*=.048) from each other. However, the late measurements taken after 10 min and 50 min of VR were not significantly different (t_35_=0.388; *P*=.99).

For disorientation difference scores, there was a significant main effect of the test period (*F*_1,35_=19.875; *P*<.001; partial *η*^2^=0.362) but no effect of the exposure duration (*F*_1,35_=2.577; *P*=.12; partial *η*^2^=0.069) and no interaction (*F*_1,35_=1.628; *P*=.21; partial *η*^2^=0.044; Figure 5).

In Table 1, a summary of one-sample *t* tests is reported to establish whether the difference scores for each measure significantly changed from zero, that is, represent a significant difference from baseline. Several visual, cognitive, and self-reported measures demonstrated changes from baseline immediately after VR; however, at the group level, all changes returned to preexposure levels at late test periods.

**Table 1:** One-sample t tests summarizing the measures that are significantly different from zero (baseline) for both test periods (immediate and late) and exposure duration.

Measure and exposure duration		Before to immediately after VR^a^			Before to 40 min (late) after VR			
		*t* test (*df*)	*P* value	Cohen *d*	*t* test (*df*)	*P* value	Cohen *d*	
**Accommodation**								
	10 min	2.824 (35)	.008	0.471	0.137	.89	0.023	
	50 min	3.709 (35)	<.001	0.618	1.804	.08	0.301	
**Convergence**								
	10 min	2.205 (35)	.03	0.367	1.172	.25	0.195	
	50 min	3.494 (35)	.001	0.582	1.324	.19	0.221	
**RT^b^ (decision)**								
	10 min	1.457 (35)	.15	0.243	−0.541	.59	−0.090	
	50 min	1.649 (35)	.11	0.275	0.602	.55	0.100	
**RT (movement)**								
	10 min	−2.725 (35)	.01	−0.454	−1.028	.31	−0.171	
	50 min	−1.206 (35)	.24	−0.201	−0.714	.48	−0.119	
**Total SSQ^c^**								
	10 min	3.427 (35)	.002	0.571	−0.627	.54	−0.105	
	50 min	4.765 (35)	<.001	0.794	0.357	.72	0.060	
**Nausea**								
	10 min	1.063 (35)	.295	0.177	−1.655	.11	−0.276	
	50 min	3.630 (35)	<.001	0.605	−0.373	.71	−0.062	
**Oculomotor**								
	10 min	3.140 (35)	.003	0.523	−0.475	.64	−0.079	
	50 min	4.639 (35)	<.001	0.773	0.094	.93	0.016	
**Disorientation**								
	10 min	3.894 (35)	<.001	0.649	1.000	.32	0.167	
	50 min	4.049 (35)	<.001	0.675	1.136	.26	0.189	

^a^VR: virtual reality.

^b^RT: reaction time.

^c^SSQ: simulator sickness questionnaire.

## Discussion

### Summary of Findings

This study aimed to investigate the consequences of playing one of the most popular active VR games for 10 min and 50 min on aspects of vision, cognition, and self-reported VR sickness. There were no dropouts due to sickness in this study. Given that the average dropout rate for a VR study using an HMD is approximately 15.6% [14], this suggests that Beat Saber was well tolerated. A low dropout rate is only one indicator of sickness, and aspects of vision, cognition, and self-reported sickness are discussed in detail in the following sections.

### Visual Measures

Irrespective of the exposure duration, both accommodation and convergence significantly changed immediately after VR compared with baseline measurements. However, the visual measures returned to baseline levels 40 min after exiting VR, suggesting that changes to accommodation and vergence were relatively short-lived.

Interestingly, the exposure duration did not influence any visual measure, suggesting that observable changes in accommodation and vergence did occur within the first 10 min of VR exposure and did not significantly change for exposures up to 50 min. Changes in convergence and accommodation observed after VR are likely to result from the perception of conflicting depth cues in HMDs. Convergence and accommodation are oculomotor functions necessary to achieve a single and clear focus on near objects, respectively. Hence, blur and disparity are essential retinotopic cues assisting accommodation and convergence to achieve a more precise fixation on a near object [51]. Typically, under natural viewing conditions, vergence and accommodation function together in a feedback loop so that changes in one mechanism will lead to concurrent changes in the other. However, in HMDs, vergence and accommodation may be decoupled [15,28], leading to an uncertainty associated with retinotopic cues for depth perception [52,53] and potentially to a range of concomitant symptoms such as headaches, sore eyes, fatigue, and double vision. Although our participants did not report any concurrent clinical visual impairments, it is worth noting that several participants reported large oculomotor changes in both immediate and late test periods (Figure 2). Large oculomotor changes after VR may influence an individual's depth perception in the real world [53], but the risks associated with these changes are not well understood.

### Cognitive Measures

In this study, we assessed whether the ability to react quickly to stimuli is affected after immersion in VR. The time required to initiate motor movements (ie, decision time) was not statistically different from the decision times before VR exposure at either test period (Figure 3). Notably, the participants were slightly faster at the late test period compared with the immediate test period. However, given that the decision times at either of these test periods were not different from their baseline measures (Table 1), this finding remains to be an interesting observation at this stage.

The investigation of the motor movement times (ie, time from button release to the touch of stimuli) revealed no concerns. In fact, after playing Beat Saber for 10 min, participants’ movement speeds immediately after VR exposure were slightly faster than before the VR exposure (Figure 3). Whether this improvement is related to practicing fast-paced motor movements required in the game remains to be established. The fact that the observed positive effects were short-lived ties in with research in the field of aerobic exercise, which found that effects of exercise on reaction times disappear quickly after exercise cessation [54]. This study cannot answer the question about the possibility of positive long-term cognitive effects with repeated VR gaming. However, there is research showing that gamers have greater attentional control compared with nongamers [55]. Furthermore, frequent gamers are better at filtering out distractors and have been shown to outperform nongamers on a range of perceptual and attentional tasks [55,56]. Considering the differences between screen-based gaming and VR gaming, it would be interesting to explore whether frequent VR gamers also exhibit a similar attentional advantage.

The literature on the immediate effects of VR exposure on the ability to react quickly to stimuli is highly inconsistent. Some researchers find negative reaction time aftereffects [19,57-59], and others show positive (faster) effects on reaction times [60]. The type of content, the time in VR, and the method of RT measurements are key reasons for the inconsistent results. Notably, although there is inconsistency across studies, the reported positive or negative effects of VR exposure on RT are typically under 50 ms. The degree to which such relatively small changes would have any real-world consequences for activities such as driving is unclear. In simulated driving studies, researchers suggest that the average braking time needs to be between 700 ms and 1200 ms to reduce the negative impact of a collision [61].

### Self-Reported VR Sickness

Self-reported VR sickness scores were higher immediately after VR than 40 min after exiting VR. Immediately after VR, participants reported more nausea, oculomotor, and disorientation symptoms compared with preexposure levels. The reported symptoms returned to preexposure levels during the late testing period.

Longer VR exposure (ie, 50 min vs 10 min) led to more symptoms immediately after VR (Figure 5). The exception was the disorientation subscale, which was not modulated by the exposure duration. An increase in nausea symptoms was observed after 50 min but not after 10 min of VR exposure (Table 1). For all other self-reported measures, 10 min of VR was sufficient to observe an increase in symptoms. All symptoms returned to baseline levels after 40 min of exiting VR (Table 1). During the late test period, the number of reported symptoms was no longer modulated by the duration of the VR exposure.

Although an increase in SSQ scores for longer exposure durations is consistent with the VR literature, few studies have examined VR sickness in HMDs, which makes it difficult to evaluate the role of intense exercise in reporting symptoms. However, considering that there is an overlap between symptoms of VR sickness and symptoms of intense cardio exercise (ie, fatigue, disorientation, sweating, and in some cases nausea), it can be challenging to identify VR sickness during an intense workout in VR. Perhaps more research with VR exergames is needed to better understand the relationship between the exposure duration, VR sickness, and the intensity of physical activity.

### Does the Lack of Prolonged Aftereffects Mean There Is Nothing to Be Concerned About?

Overall, this study found no strong evidence for adverse symptoms of concern 40 min after VR exposure, irrespective of whether people played Beat Saber for 10 or 50 min. However, our findings should *not* be taken as evidence for a clean bill of health for playing VR exergames. Primarily, our participants comprised a young and healthy student population with a below-average history of motion sickness. Hence, it is unclear whether the current findings will hold for the elderly and people with high susceptibility to motion sickness. It is important to keep in mind that the lack of difference between baseline scores and 40 min after VR is based on group averages. Closer inspection of individual data (Figures 2 and 5) reveals that individual participants still report adverse symptoms that are higher than their baseline scores. Case in point, approximately 1 out of 7 participants (approximately 14%) may still score *high* on the SSQ 40 min after playing Beat Saber for 50 min (Figure 4). The *high* category in this study is based on the study by Kennedy et al [49], who proposed that scores over 20 are indicative of a problem simulator. However, the degree to which a high SSQ score impacts everyday activities is unclear. The link between scores on the SSQ and real-world performance decrements is not understood and constitutes a critical gap in knowledge for advancing the safe use of VR.

We did not follow up with participants after the late measurements and did not know how symptoms developed after the experimental session ended. It is important to note that the peak of symptoms in an individual is not always observed in or immediately after VR. Some users may experience severe latent symptoms occurring up to 24 h later [30,36,62]. There are many challenges with monitoring participants’ hours after a VR experiment such as how long should one follow up with participants, what is the best method for measuring latent effects, and should symptoms be weighted higher if they occur hours after VR. Furthermore, when following up with participants’ hours later, it is more difficult to establish a causal relationship between their symptoms and VR. For example, headaches and fatigue are symptoms of VR sickness but can also occur for many other reasons not related to VR, that is, dehydration, hunger, and exertion, making it difficult to track the progression of symptoms for each individual.

Individual differences such as age, HMD fit, postural stability, and motion sickness susceptibility may contribute to the likelihood of a person experiencing VR sickness in HMDs [14,63,64]. The alluvial plot (Figure 5) shows subgroup patterns on the relationship between the levels of symptoms and the exposure durations immediately after exiting. When experiencing a high level of symptoms after a short 10-min exposure, a participant was almost certain to also experience a high level of symptoms for a longer 50-min exposure on a different day. Around half of the participants experiencing midlevels or no/low symptoms immediately after a 10-min exposure reported the same level of symptoms after longer exposure durations. The other half of the participants in these categories experienced worse symptoms for a longer 50-min exposure. Experiencing symptoms after short periods in VR may be an indicator of whether an individual may experience serious symptoms after longer exposure periods. There is some evidence to suggest that short repeated exposures may reduce a user's experience of VR sickness in subsequent exposures [65-67], but this strategy may not work for everyone. Additionally, taking more frequent breaks [31-33] could be explored as a strategy to mitigate symptoms.

### Limitations

We used the currently most popular VR exergame; however, to what extent these findings hold for other games is unknown. One major challenge when comparing aftereffects from different VR experiences is the role content has on the progression and severity of symptoms. For example, several studies have compared sickness symptoms across multiple VR experiences and found that some content induces VR sickness and other content leads to no/minimal symptoms [37,59]. High levels of motion have consistently been shown to increase nausea, disorientation, and oculomotor disturbances [21,37,58]. Other factors such as scene complexity [68], presence [10,11], and locomotion [69] have also been suggested to play a role in the development of VR sickness.

In our Beat Saber study, there was a high level of both visual stimulation and user movement, which would have increased with the level difficulty. Participants were able to select any song or level of difficulty. By directing their own gameplay, participants could choose which levels they felt challenged by. A limitation of this study is that we did not monitor in-game performance, which may have been insightful. If the choice of difficulty increased the visual motion in the exergame, this may have also had an impact on the likelihood of a person experiencing symptoms. Future VR studies should consider how the variation in gameplay may impact sickness outcomes and measure in-game performance if possible.

VR exergames that require physical activity from the user will have higher levels of user movement and visual stimulation, which may contribute to VR sickness. A recent VR bike simulator study [57] found that participants using an HMD displayed substantially higher SSQ scores than participants using a large screen. In their study, SSQ scores increased with the exposure duration and simulated motion [57]. Although HMDs may be a more immersive option for exergames, HMDs may also lead to higher levels of sickness relative to screen-based exergames. If the simulated motion is a major factor leading to VR sickness in exergames, then congruent visual and user motion will likely be better tolerated. A call for more research is needed to investigate the relationship between simulated motion and VR sickness in exergames.

A wide range of possible VR aftereffects exist, and this study only investigated some of them (vision, reaction time, and self-reports). It remains to be established whether or how other symptoms, such as changes in balance, depth perception, and motion drowsiness (sopite symptoms), may have also been affected. This study also targeted a young and healthy group of participants, and it is possible that an older sample may experience different symptoms or challenges when exergaming. There are no clear guidelines and thresholds identifying who will be at risk after using provocative VR applications and what to do if users experience atypical symptoms.

### Conclusions

VR sickness in exergames, particularly in HMDs, is understudied. On the basis of the data in this study, we can make two suggestions for the safe use of VR. First, we recommend that users trial a brief session in VR before committing to longer exposures. Our research shows that if a user experiences a high level of symptoms after a short exposure, they he or she will likely experience similar or worse symptoms for longer exposures. Users with high symptoms for shorter exposures may attempt to take frequent breaks and habituation strategies but should still be cautious as these approaches may not work for everyone. Second, we recommend that users commit to a waiting period after exiting VR. During this waiting period, users should withdraw from activities that may pose a risk to injury in the event that a person has VR aftereffects, for example, driving a car. In our study, a 40-min wait period was sufficient for most people's symptoms to return to baseline levels. Since content plays a major role in the development of VR sickness, more research is needed to clarify the relationship between VR sickness, the exposure duration, and the minimum waiting times for different exergames before participants should return to activities that pose an increased risk of injury and accident. Given the increasing popularity of VR exergames and the potential implications of VR aftereffects, it is essential that research in this area continues to propagate.

## Abbreviations

HMD: head-mounted display
RAF: Royal Air Force
RT: reaction time
RTI: reaction time task
SSQ: Simulator Sickness Questionnaire
VR: virtual reality
